# Low-level laser prevents doxorubicin-induced skeletal muscle atrophy by modulating AMPK/SIRT1/PCG-1α-mediated mitochondrial function, apoptosis and up-regulation of pro-inflammatory responses

**DOI:** 10.1186/s13578-021-00719-w

**Published:** 2021-12-07

**Authors:** Hsiu-Chung Ou, Pei-Ming Chu, Yu-Ting Huang, Hui-Ching Cheng, Wan-Ching Chou, Hsin-Lun Yang, Hsiu-I. Chen, Kun-Ling Tsai

**Affiliations:** 1 Department of Physical Therapy, College of Medical and Health Science, Asia University, Taichung, Taiwan, ROC; 2 Department of Anatomy, School of Medicine, China Medical University, Taichung, Taiwan, ROC; 3 Department of Physical Therapy, College of Medicine, National Cheng Kung University, Tainan, Taiwan, ROC; 4 Institute of Allied Health Sciences, College of Medicine, National Cheng Kung University, Tainan, Taiwan, ROC; 5 Department of Physical Therapy, Hungkuang University, Taichung, Taiwan, ROC

**Keywords:** Low-level laser, Doxorubicin, Myopathy, ROS, AMPK

## Abstract

**Background:**

Doxorubicin (Dox) is a widely used anthracycline drug to treat cancer, yet numerous adverse effects influencing different organs may offset the treatment outcome, which in turn affects the patient’s quality of life. Low-level lasers (LLLs) have resulted in several novel indications in addition to traditional orthopedic conditions, such as increased fatigue resistance and muscle strength. However, the mechanisms by which LLL irradiation exerts beneficial effects on muscle atrophy are still largely unknown.

**Results:**

The present study aimed to test our hypothesis that LLL irradiation protects skeletal muscles against Dox-induced muscle wasting by using both animal and C2C12 myoblast cell models. We established SD rats treated with 4 consecutive Dox injections (12 mg/kg cumulative dose) and C2C12 myoblast cells incubated with 2 μM Dox to explore the protective effects of LLL irradiation. We found that LLL irradiation markedly alleviated Dox-induced muscle wasting in rats. Additionally, LLL irradiation inhibited Dox-induced mitochondrial dysfunction, apoptosis, and oxidative stress via the activation of AMPK and upregulation of SIRT1 with its downstream signaling PGC-1α. These aforementioned beneficial effects of LLL irradiation were reversed by knockdown AMPK, SIRT1, and PGC-1α in C2C12 cells transfected with siRNA and were negated by cotreatment with mitochondrial antioxidant and P38MAPK inhibitor. Therefore, AMPK/SIRT1/PGC-1α pathway activation may represent a new mechanism by which LLL irradiation exerts protection against Dox myotoxicity through preservation of mitochondrial homeostasis and alleviation of oxidative stress and apoptosis.

**Conclusion:**

Our findings may provide a novel adjuvant intervention that can potentially benefit cancer patients from Dox-induced muscle wasting.

**Supplementary Information:**

The online version contains supplementary material available at 10.1186/s13578-021-00719-w.

## Introduction

Cancer has been considered one of the top leading causes of mortality worldwide for decades, and pharmacological intervention strategies rely heavenly on chemotherapy to treat cancer patients. Doxorubicin (Dox), an anthracycline antibiotic, has been widely used in the treatment of hematological malignancies and solid tumors for five decades [1]. However, its clinical use in higher or more effective doses is restricted due to the possibility of developing severe toxicity in several organs in human systems [2]. The antineoplastic effects of Dox are mainly attributed to its properties of interrupting DNA replication as well as RNA transcription. Because of interfering with the synthesis of DNA, RNA, and proteins, the division of cancerous cells is effectively abolished. Due to its nonspecific mechanism of action, surrounding healthy cells with highly proliferative characteristics are inevitably damaged. Clinically, cachexia presenting with nausea, fatigue, hair and weight loss, skeletal muscle atrophy, and cardiotoxicity together negatively impact the quality of life of patients when administered systemically. Therefore, adjuvant therapy that may mitigate cachexia due to chemotherapy is of considerable clinical value.

To date, the potential molecular mechanisms of doxorubicin (Dox)-induced muscle atrophy have attracted extensive attention. Previous experiments found compelling evidence suggesting that doxorubicin can cause muscle atrophy due to autophagy followed by oxidative stress [3], a reduction in mitochondrial content and reactive oxygen species (ROS) production [4], lesions in the electron transport system of mitochondria [5], disruption of mitochondrial energy metabolism and redox balance [6], and activation of apoptosis and the ubiquitin–proteasome pathway [7]. Therefore, further understanding of these mechanisms is needed to develop an adjuvant therapy to lessen the adverse effects caused by Dox treatment.

AMP-activated protein kinase (AMPK), a pivotal sensor of cellular energy levels, is a serine-threonine kinase that plays a key role in facilitating glucose uptake in skeletal muscles, which are composed of more than half of the body weight in humans and account for nearly 75% of the insulin-regulated glucose uptake in our bodies. Decreased AMPK activity has been considered a negative side effect of Dox treatment. Earlier research demonstrated that Dox treatment induced a significant impairment in glucose uptake by AMPK inhibition [8]. Furthermore, Dox increased systemic insulin resistance [9], which is mediated by inhibition of AMPK signaling in skeletal muscles [8]. In contrast, activated AMPK can improve cellular energy status and maintain mitochondrial function [10]. Meanwhile, AMPK regulates the activity of Sirtuin 1 (SIRT1) and increases intracellular NAD^+^, which can activate NAD^+^-dependent SIRT1 to elicit biological effects. Activated AMPK and SIRT1 regulate the activity of coactivator peroxisome proliferator-activated receptor coactivator-1α (PG-1α), further upregulating its expression [11]. AMPK phosphorylation of PGC-1α initiates many of the important gene regulatory functions of AMPK in skeletal muscle [12]. Moreover, the AMPK/SIRT1/PGC-1α signaling pathway acts as an energy-sensing network and plays a crucial regulatory role in mitochondrial biosynthesis, energy metabolism, and oxidative stress [11]. Research previously showed that Dox treatment inhibits AMPK and PGC-1α signaling pathways, resulting in intensifying myocardial injury [13]. Accordingly, interventions that can activate AMPK may have the potential to mitigate the negative effects on the muscle wasting caused by Dox treatments [14].

ROS possess multiple functions, such as activation of mitogen-activated protein kinases (MAPKs) and translocation of nuclear factor (NF)-κB from the cytosol to the nucleus, and both of these functions are known as key regulators of various pathological processes. In particular, P38 MAPK can be activated by ROS, which in turn triggers muscle wasting [15]. On the other hand, excessive ROS are also known to result in disruption of the outer mitochondrial membrane, which is accompanied by an increase in mitochondrial membrane permeability. Consequently, a variety of proteins and proteases within the mitochondria-related to apoptosis are released into the cytosol, which in turn triggers apoptosis by cleaving cellular proteins, thereby accelerating the progression of cell death. Excessive ROS are involved in Dox-induced myopathy, which is oxidative-induced [16]. For this reason, interventions that can reduce oxidative damage in skeletal muscles by suppressing the ROS-mediated MAPK/NF-κB signaling pathway and mitochondrial dysfunction are assumed to be clinically beneficial [15].

Low-level laser (LLL) irradiation has been shown to modulate a wide range of biological processes. Traditionally, LLL is commonly used in orthopedic conditions, such as wound healing and inflammation. In recent years, additional novel application areas have been intensively discovered. For example, these include increased muscle fatigue resistance [17], reduced post fatigue concentrations of serum lactate and creatine kinase levels [18], increased exercise performance, decreased exercise-induced oxidative stress and muscle damage [19], and improved muscle performance in elderly people [20]. Similarly, LLL can effectively promote the recovery of gastrocnemius skeletal muscle from atrophy in rats [21], accelerate muscle regeneration in aged rats [22], and increase mitochondrial membrane potential and ATP synthesis in skeletal cells [23]. In terms of cellular mechanisms, LLL has been reported to increase mitochondrial respiration, which leads to an enhancement of ATP production [24]. In addition, LLL is capable of reestablishing cellular homeostasis, stimulating the mitochondrial respiratory chain, and increasing adenosine triphosphate (ATP) production and the synthesis of proteins and enzymes [25]. Since mitochondria are thought to be the principal photoacceptors present inside cells and mitochondria are extraordinarily distributed in muscle cells, LLL irradiation is supposed to be highly beneficial for muscle damage. Collectively, we assume that LLL potentially exerts protective effects on skeletal muscle and delays the progression of Dox-induced atrophy.

In the present study, we first examined the benefits of LLL on Dox-induced myopathy in both animal and myoblast cell culture models. The AMPK/SIRT1/PGC-1α signaling pathways, oxidative stress, mitochondrial function, and apoptosis-related parameters that were activated by Dox were explored; both in vivo and in vitro studies were conducted to elucidate the cellular and molecular mechanisms underlying LLL's beneficial effects.

## Materials and methods

### Cell culture and reagents

C2C12 skeletal myoblast cells (ATCC CRL-1772, USA) were grown in Dulbecco's modified Eagle's medium (DMEM; Gibco, NY, USA) supplemented with fetal bovine serum (FBS) to a final concentration of 10%. The cells were passaged every second day, and confluency was maintained below 80% to prevent spontaneous differentiation. First, 5,58,6,68 tetraethylbenzimidazolcarbocyanine iodide (JC-1), EX-527, 2',7' –dichlorofluorescein diacetate (DCF-DA) and MitoSox reagent were obtained from Thermo Scientific (Waltham, MA, USA). MitoQ, SB203580, penicillin, and streptomycin were purchased from Sigma (St. Louis, MO, USA). Anti-β-actin, anti-p38, anti-p-p38, anti-NF-kBp65, anti-HDAC1, and anti-caspase-3 antibodies were all obtained from Santa Cruz Biotechnology (Santa Cruz, CA, USA). Anti-p-AMPK, anti-SIRT1, anti-PGC-1α, anti-Bax, anti-Bcl2, HRP-conjugated anti-rabbit, and anti-mouse secondary antibodies were purchased from Transduction Laboratories (CA, USA).

### Animal care and experimental groups

**Fig. 1:**
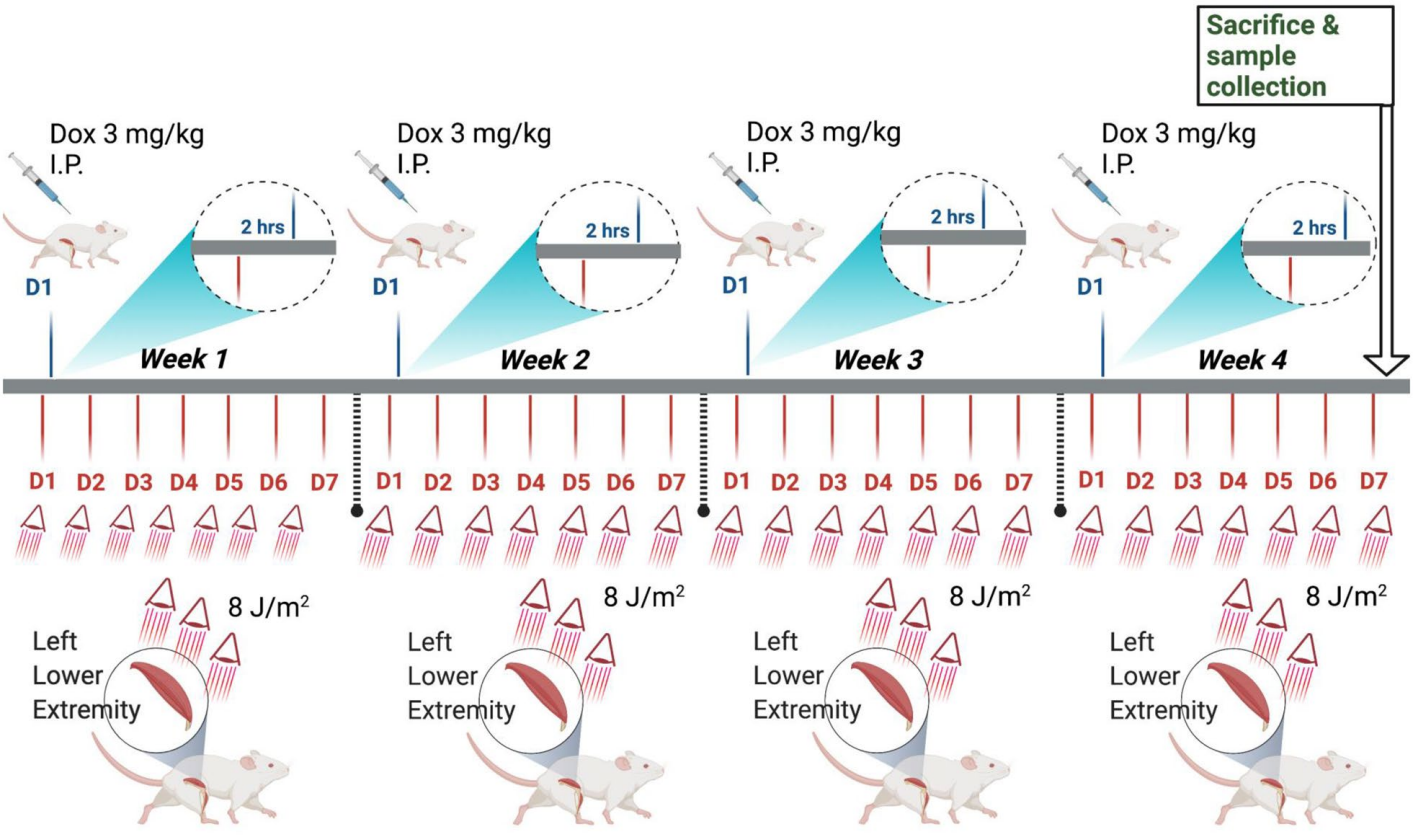
Schematic diagram of the animal experimental. Animals were allocated to three groups: Control group (Ctl), Dox-receiving group (Dox), and LLL treatment group (Dox + LLL). In Dox-treat animals, Dox (3 mg/kg) was injected by intraperitoneal injection per week ( total 4 weeks study period, accumulated dosage: 12 mg/ kg). In LLL-exposed animals, the laser was applied 10 min per point, 1 time per day, and 7 days per week. On the first day of the 1st, 2nd, 3rd and 4th week, after 2 h of LLL irradiation, Dox was injected by i.p. injections. Samples were collected on the last day of this 4-weeks experimental

In this study, 16 male Sprague Dawley (SD) rats were placed in plastic cages and kept in a room with controlled environmental conditions with access to water and standard food. The experimental procedures were approved by the Ethics Committee of Cheng Kung University, Tainan, Taiwan, and conducted in accordance with the ‘Guide for Care and Use of Laboratory Animals’. Two groups of rats underwent the following treatments: control (saline) and Dox, with n = 16 (8 in each group). Dox was prepared in sterilized distilled water and administered at 3 mg/kg body weight (BW) via 4 intraperitoneal (i.p.) injections weekly, giving a cumulative dose of 12 mg/kg BW. Control rats received three i.p. injections of 0.9% saline on alternative days. In the Dox group, each rat received 8 J/m^2^ LLL irradiation on the left (L’t), but not on the right (R’t), of the low extremity calf muscles before Dox injection. LLL irradiation (8 J/m^2^) was continually administered to the L’t lower extremities for a duration of 4 weeks after Dox injection. During LLL treatment, animals were anesthetized intramuscularly using a mixture of 10:1 tiletamine/zolazepam (Zoletil) (Virbac, Carros, France) and xylazine (Rompun) (Bayer, Pittsburgh, PA, USA). The dosage of anesthesia was 0.1 mL Zoletil/100 g body weight. Animals were sacrificed 7 days after the last injection of Dox after anesthetization with 4% isoflurane via a nose cone. The protocol of this animal study is presented in Fig. 1. All of the animal investigations followed guidelines that were required for the care and use of laboratory animals and were approved by the animal center of the National Cheng Kung University in Tainan, Taiwan (Approval No. 109077). Soleus muscles on both legs were harvested and either flash-frozen for RNA and protein analysis as well as TUNEL staining or kept in 4% paraformaldehyde (Thermo Fisher Scientific, Waltham, MA, USA) for histological staining.

### LLL

An AlGaInP-diode laser (AM-800, Konftec Co., Taipei, Taiwan) was used for in vivo and in vitro investigations. For in vitro studies, the probe of the laser was fixed vertically 15 cm above the cells. Laser irradiation was applied at a wavelength of 660 nm for 10 min (8 J/cm^2^). After 1 h of LLL irradiation, 10 µM Dox was added to cells for 24 h stimulation. For in vivo studies, the laser was applied over the muscle belly and then irradiated every 1 cm for treatment of 3 points in the central, proximal, and distal areas of the muscle belly, with a treatment point of 1 cm^2^, 10 min per point, 1 time per day, and 7 days per week. On the first day of the 1st, 2nd, 3rd and 4th week, after 2 h of LLL irradiation, Dox was injected by i.p. injections.

### Hematoxylin and Eosin (H&E) staining

Histology was performed to determine the myofibrillar size and atrophy, as described previously [26]. Transverse soleus sections were stained with H&E (Thermo Fisher Scientific), and representative images were taken at 100 × magnification.

### RNA isolation and analysis

RNA was isolated from frozen muscle using 1 ml of TRIzol reagent (Invitrogen, Carlsbad, CA, USA) according to the manufacturer’s instructions. The extracted RNA was dissolved in Tris–HCl and ethylene-diamine tetraacetic acid (pH 7.6) and quantified spectrophotometrically. The purity was assessed by determining the ratio of the absorbance at 260 nm and 280 nm. All samples had 260–280-nm ratios above 2.0. The integrity of the RNA was confirmed by inspection of ethidium bromide-stained 18S and 28S ribosomal RNA under ultraviolet light (Invitrogen, Carlsbad, CA, USA).

### Reverse transcription

Total RNA was reverse transcribed into cDNA in two steps. In the first step, 1 μl of oligo (dT) primer (Invitrogen, Carlsbad, CA, USA) and 9.5 μl of water were added to 1 μg of isolated total RNA, heated to 70 °C for 10 min and then immediately cooled on ice. In the second step, 4 μl of 5 × reverse transcription buffer, 1 μl of a dNTP (Promega, Madison, WI, USA) mixture containing 0.2 mM each of dATP, dCTP, dGTP, and 0.1 M dTTP, 2 μl of 0.1 M dithiothreitol, and 0.5 μl of M-MLV RT enzyme (Promega, Madison, WI, USA) in a total volume of 20 μl were combined; the total mixture was incubated at 42 °C for 60 min for each sample. To minimize any potential variations in the reverse transcription reaction, all RNA sample groups were reverse transcribed simultaneously. The primers used are listed in Additional file 1: Table S1. The threshold cycle (Ct) values were normalized by the Ct value calculated for β-actin.

### Analysis by real-time polymerase chain reactions

The RNA transcript levels for the different experimental and control muscles were analyzed simultaneously, and the reactions were carried out in duplicate using SYBR green fluorescent dye (Applied Biosystems, Foster City, CA, USA) in a sequence detection system (GeneAmp 5700; Applied Biosystems).

### Measurement of mitochondrial membrane potential

The lipophilic cationic probe fluorochrome 5,58,6,68 tetraethylbenzimidazolcarbocyanine iodide (JC-1) was used to explore the effects of Dox on mitochondrial membrane potential (ΔΨm). JC-1 exists either as a green-fluorescent monomer at depolarized membrane potentials or as a red fluorescent J-aggregate at hyperpolarized membrane potentials. After treating the cells with Dox (2 μM) for 24 h in the presence or absence of CGA, cells were rinsed with M199 and then loaded with JC-1 (5 μM). After a 30-min incubation at 37 °C, cells were examined by flow cytometry.

### Biogenesis of mitochondria

Mitochondrial mass was tested using N-nonyl acridine orange (NAO) staining. Endothelial cells were stained with NAO (5 μM) in the dark for 30 min at 37 °C, and the cells were examined by flow cytometry. Real-time PCR was used to test cellular and mitochondrial DNA (mtDNA) content. The following primers were designed to investigate cytochrome b: sense primer 5′-TTTGGGTCCCTTCTAGGAGTC-3′ and antisense primer 5′-CCGACATGAAGGAATAAGCAA-3′. PCR was performed using SYBR Green on an ABI 7000 sequence detection system (Applied Biosystems) according to the manufacturer’s instructions.

### Measurement of mitochondrial ROS production

ROS generation in the mitochondria of the C2C12 cells was determined using MitoSOX. C2C12 cells (10^4^ cells per well) in 96-well plates received LLL irradiation at an intensity of 8 J/m^2^ for 2 h. Afterward, 2 μM Dox was added to the medium for an additional 24 h. After removing the medium from the wells, cells were then incubated with 10 μM MitoSOX or 10 μM DCF-DA for or 30 min. The fluorescence intensity was calculated by flow cytometry.

### Transfection with small interfering RNA (siRNA)

On-target Plus SMART pool siRNAs for the nontargeting control and SIRT1, AMPK-α, and PGC-1α were purchased from Dharmacon. Two days after transfection, cells were treated with a reagent as indicated for further experiments.

### Immunoblotting

At the end of Dox exposure, endothelial cells were washed, scraped from dishes, and lysed in RIPA buffer. Proteins were then separated by electrophoresis on an SDS–polyacrylamide gel. After the proteins were transferred to a PVDF membrane, the blot was incubated with blocking buffer for 1 h at room temperature, probed with primary antibodies overnight at 4 °C, and then incubated with horseradish peroxidase-conjugated secondary antibody for 1 h.

### Terminal deoxynucleotidyl transferase (TdT) dUTP nick end labeling assay

All muscle tissues were fixed in 10% neutral buffered formalin and embedded in paraffin. Investigation of apoptotic cells was performed using the In Situ Cell Death Detection Kit (11,684,795,910; Roche) following the manufacturer’s direction. The TUNEL Colorimetric Detection Kit (C10625, Invitrogen) was used for tissue study. Streptavidin conjugated horseradish peroxidase (HRP) was then bound to the biotinylated nucleotides, and visualized using the peroxidase substrate, 3,3’-diaminobenzidine tetrachloride (DAB). The nuclei of apoptotic cells could be observed in brown color under light microscope. The apoptotic index was calculated by dividing the number of TUNEL-positive cells though the total number of cells in the field.

### Investigation of mitochondrial oxygen consumption

The mitochondrial oxygen consumption rate (OCR) was measured by the Seahorse analyzer (Seahorse, USA). C2C12 were seeded at 10,000 cells per well in a 96-well XF culture plate. At the beginning of mitochondrial OCR measurement, culture medium was replaced with the commercial XF assay medium and incubated for 1 h in a CO2-free incubator. The mitochondrial OCR was analyzed using version 2.6 of the Seahorse Wave software.

### Statistical analyses

Results are expressed as the means ± SD. Statistical analyses were performed using one-way or two-way ANOVA, followed by Tukey’s test as appropriate. A *P*-value < 0.05 was considered statistically significant.

## Results

### LLL irradiation ameliorates Dox-induced muscle wasting in animals

**Fig. 2:**
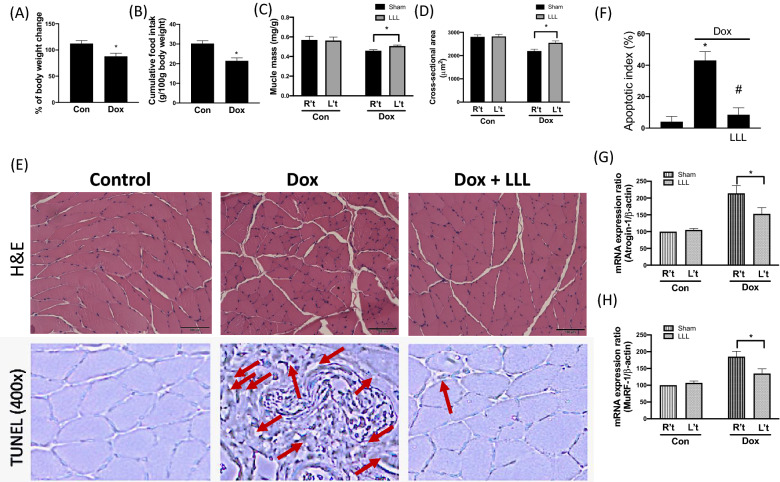
LLL exposure reverse Dox-induced muscle wasting in animals. The effects of LLL on Dox-induced muscle atrophy. The weight change (**A**), food intake (**B**), muscle mass (**C**), and cross-sectional area (**D**) in Con and Dox animals. Representative hematoxylin–eosin (H&E) and TUNEL stained soleus (**E**). The arrow heads indicated TUNEL ( brown color). Quantification of the percentage of TUNEL positive nuclei (**F**). Atrogin-1 (**G**) and MuRF-1 (**H**) mRNA expression levels in soleus muscle were investigated by real-time PCR. The data were presented as the mean ± SD of 8 animals. (* indicating p < 0.05 compared with the control group)

First, we observed changes in body weight, cumulative food intake, muscle mass, and the cross-sectional area between the two experimental groups. As shown in Fig. 2A and B, body weight and cumulative food intake were significantly lower 28 days after the first injection in rats than in the control group. We further assessed the differences in soleus muscle mass and cross-sectional area individually between the right leg and left leg; the latter received LLL irradiation in the Dox-injected group. We found that LLL irradiation markedly restored the decreased soleus muscle mass and cross-sectional area (Fig. 2C, D). To further confirm the effects of LLL irradiation on the Dox-induced changes in the skeletal muscle architecture, slices of soleus muscles stained with hematoxylin and eosin (H&E) showed normal architecture with normal interstitial space in the control group. In contrast, abnormal architecture and enlarged interstitial spaces were observed in the right legs of the Dox-injected group, whereas these abnormalities were reversed in the left legs that received LLL irradiation. In addition, a similar tendency was found in TUNEL staining, which presented the number of apoptotic cells (Fig. 2E, F). Prior research has indicated that Atrogin-1 and MuRF-1 are important regulators of skeletal muscle degradation [7]; therefore, we attempted to ascertain whether Atrogin-1 and MuRF-1 are involved in the beneficial effects of LLL irradiation on Dox-induced muscle wasting. The results from mRNA analyses showed that the expression of Atrogin-1 and MuRF was much higher in the Dox-injected group than in the control group (Fig. 2F, G). The expression levels in the left legs, which received LLL irradiation, were significantly reduced, suggesting that LLL irradiation protects against Dox-induced muscle atrophy through, at least in part, diminishment of ubiquitin-mediated protein degradation in skeletal muscles. In addition,

### LLL reverse Dox-impaired AMPK phosphorylation

**Fig. 3:**
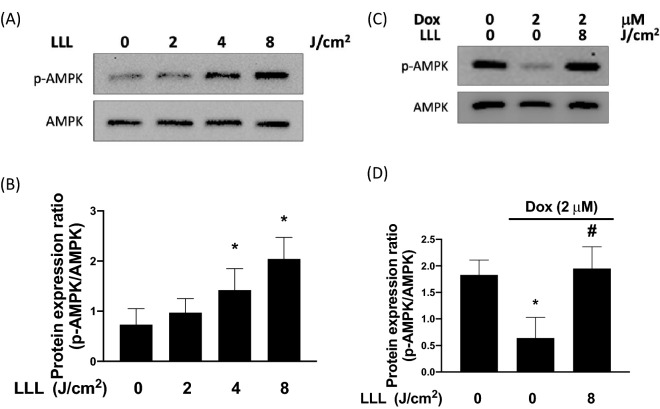
Low level laser (LLL) exposure increases the phosphorylation of AMPK. C2C12 cells were exposed to various dosages of LLL for a total of 2 h. The expression levels of phosphorylated AMPK and total AMPK were investigated using Western blot assay (**A**). Protein expression levels were quantified and presented by a bar chart (**B**). C2C12 cells were treated with 2 µM doxorubicin (Dox) for 24 h, in the LLL-treated group, cells were exposed to LLL 8 J/m^2^ before Dox treatment. The expression level of phosphorylated AMPK and total AMPK were investigated using Western blot assay (**C**). Protein expression levels were quantified and presented by a bar chart (**D**). The data were presented as the mean ± SD of three biological replicates at three separate times. (* indicating p < 0.05 compared with the control group; ^#^indicating p < 0.05 compared to Dox group)

Subsequently, we wanted to further explore the molecular mechanisms of the protective effects of LLL irradiation on Dox-induced muscle atrophy. Since AMPK activation plays a crucial role in alleviating mitochondrial oxidative damage in cardiotoxicity caused by Dox [27], we aimed to investigate whether AMPK activation is involved in the protective effects of LLL irradiation by using C2C12, a myoblast cell line. We first determined the effects of LLL irradiation on the phosphorylation levels of AMPK in terms of different intensities. As shown in Fig. 3A and B, our results demonstrated that the phosphorylation levels of AMPK were increased in cells receiving LLL irradiation for 1 h in a dose-dependent manner (2–8 J/m^2^). Accordingly, 8 J/m^2^ of intensity and a 1-h irradiation period were selected for the following experiments. After treatment with 2 μM of Dox in C2C12 cells for 24 h, the phosphorylation of AMPK was markedly reduced compared to that in the control cells. However, the level of phosphorylation was restored to nearly normal in cells exposed to LLL prior to treatment with Dox (Fig. 3C, D).

### LLL reverses dox-induced dephosphorylation of AMPK by modulating SIRI1 and PGC-1α

**Fig. 4:**
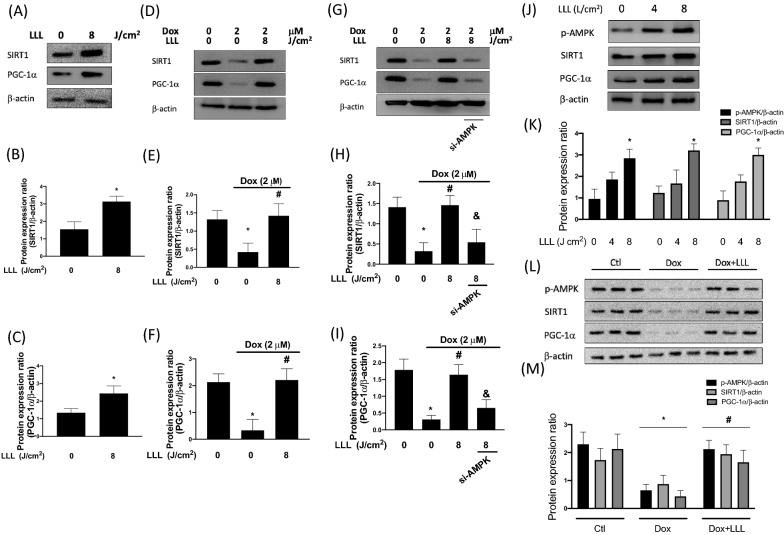
LLL exposure mitigates Dox-reduced SIRT1 and PGC-1α expression via modulation of AMPK. C2C12 cells were exposed to LLL 8J/m^2^ for 2 h and incubated in the 37 °C incubator with 5% CO2 for additional 22 h. The expression level of SIRT1, PGC-1α, and β-actin were investigated using Western blot assay (**A**). Protein expression levels were quantified and presented by a bar chart (**B**, **C**). C2C12 cells were treated with 2 µM doxorubicin (Dox) for a total of 24 h, in the LLL-treated group, cells were exposed to LLL 8 J/m^2^ before Dox treatment. The expression level of SIRT1, PGC-1α, and β-actin were investigated using Western blot assay (**D**). Protein expression levels were quantified and presented by a bar chart (**E**, **F**). In the LLL-treated group, cells were exposed to LLL 8J/m^2^ for 2 h before Dox treatment. In LLL plus AMPK silencing group, cells were transfected with AMPK siRNA for 36 h before LLL exposure and Dox treatment. The expression level of SIRT1, PGC-1α, and β-actin were investigated using Western blot assay (**G**). Protein expression levels were quantified and presented by a bar chart (**H**, **I**). Western blot analysis of phosphorylated AMPK, SIRT1, and PGC-1α in the left soleus muscle from after LLL intervention without Dox injection (**J**). Bar graphs illustrate densitometric analyses of phosphorylated AMPK, SIRT1, and PGC-1α expression levels (**K**). Western blot analysis of phosphorylated AMPK, SIRT1, and PGC-1α in the left soleus muscle (**L**). Bar graphs illustrate densitometric analyses of phosphorylated AMPK, SIRT1, and PGC-1α expression levels (**M**). The data were presented as the mean ± SD of three biological replicates at three separate times. (* indicating p < 0.05 compared with the control group; # indicating p < 0.05 compared to Dox group; & indicating p < 0.05 compared to only LLL exposure group)

AMPK can switch and regulate various intracellular signals to protect cells from oxidative stress. The downregulated AMPK/SIR1/PGC-1α signaling pathway leads to muscle atrophy in aging rats [28]. Therefore, we investigated the effects of LLL irradiation on the expression levels of SIRT1 and PGC-1α. We revealed that LLL irradiation 1 h followed by 23 h incubation up-regulates SIRT1 and PGC-1α expression levels (Fig. 4A–C). Next, we examined the effects of LLL irradiation on the expression levels of SIRT1 and PGC-1α after treatment with 2 μM of Dox for 24 h. Our data in Fig. 4D–F suggest that LLL irradiation reversed the Dox-mediated downregulation of SIRT1 and PGC-1α expression in skeletal muscle cells. We subsequently confirmed the involvement of AMPK in the signaling pathway by siAMPK. We found that the beneficial effects of LLL irradiation on the expression levels of SIRT1 and PGC-1α were diminished in AMPK knockdown cells. These results indicated that LLL mitigated Dox-downregulated SIRT1 and PGC-1α expression through the activation of AMPK (Fig. 4G–I). In animal model, we revealed that LLL up-regulated AMPK/SIR1/PGC-1α pathway in a dosage-dependent manner (Fig. 4J, K). The protective effect of LLL on Dox-repressed downregulated AMPK/SIR1/PGC-1α signaling pathway had been further confirmed in animals (Fig. 4L, M).

### LLL protects against Dox-impaired mitochondrial function and oxidative stress by modulating the AMPK/SIRT1/PGC-1α pathway

**Fig. 5:**
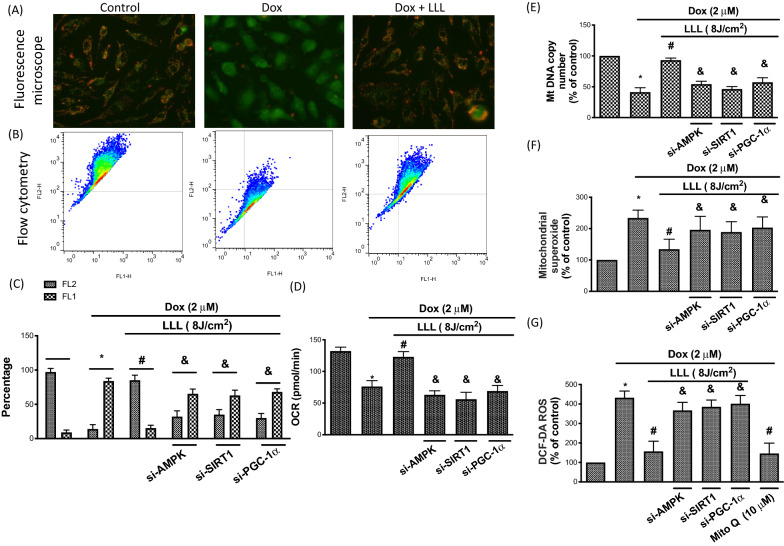
LLL exposure protects against Dox-impaired mitochondria function and biogenesis. C2C12 cells were treated with 2 µM doxorubicin (Dox) for a total of 24 h, in the LLL-treated group, cells were exposed to LLL 8 J/m^2^ before Dox treatment. In LLL plus AMPK, SIRT1, and PGC-1α silencing groups, cells were transfected with AMPK or SIRT1 or PGC-1α siRNA for 36 h before LLL exposure and Dox treatment. The mitochondrial membrane potential was investigated by JC-1. Results of JC-1 were obtained by a fluorescence microscope (**A**) and flow cytometry (**B**). FL-1 (green) and FL-2 (red) from the flow cytometry were quantified and presented by a bar chart (**C**). The mitochondrial oxygen consumption rate (ORC) was investigated by the Seahorse analyzer (**D**). The mt DNA copy number, which represents the biogenesis of mitochondrial, was investigated by the real-time PCR (**E**). The mitochondria superoxide was tested using the MitoSOX (**F**). Cytosolic ROS was investigated by DCF-DA (**G**). The data were presented as the mean ± SD of three biological replicates at three separate times. (* indicating p < 0.05 compared with the control group; # indicating p < 0.05 compared to Dox group; & indicating p < 0.05 compared to only LLL exposure group)

Prior research indicated that Dox induces atrophy partly by causing mitochondrial dysfunction in skeletal muscles [6]. We, therefore, measured the mitochondrial membrane potential (ΔΨm) by flow cytometry using JC-1 dye. Our data demonstrated that LLL irradiation 1 h before treatment with Dox attenuated Dox-induced mitochondrial membrane potential depolarization by fluorescence microscopy (Fig. 5A) and flow cytometry (Fig. 5B). However, the cytoprotective effects of LLL irradiation were eliminated in cells with silenced SIRT1, AMPK, and PGC-1α by siRNA transfection (Fig. 5C). We also found that LLL irradiation before Dox stimulation up-regulated Dox-reduced mitochondrial oxygen consumption rate (OCR), this finding was reversed by SIRT1, AMPK, and PGC-1α by siRNA transfection (Fig. 5D). To further determine whether LLL irradiation enhanced mitochondrial biogenesis and ROS generation, the contents of mitochondrial DNA (MtDNA) and superoxide generated by mitochondria were measured. Our results suggested that MtDNA content was reduced and superoxide was increased in cells treated with Dox, and LLL significantly reversed these changes. However, cells treated with Dox along with AMPK, SIRT1, and PGC-1α siRNA transfection abolished the beneficial effects of LLL irradiation (Fig. 5D, E). The mitochondria impairment triggers cytosolic oxidative stress [29]. We displayed that Dox increased the cytosolic ROS by DCF-DA assay, LLL reduced cytosolic ROS level. In addition, Mito Q inhibited Dox-upregulated cytosolic ROS, indicating that Dox elevates cytosolic ROS through increasing mitochondria oxidative stress (Fig. 5F). Our data indicated that LLL prevents Dox-induced mitochondria impairment, thereby mitigating Dox-mediated cytosolic ROS elevation.

### LLL reduces Dox-induced P38 MAPK phosphorylation and NF-kB activation via a ROS-dependent signaling pathway

**Fig. 6:**
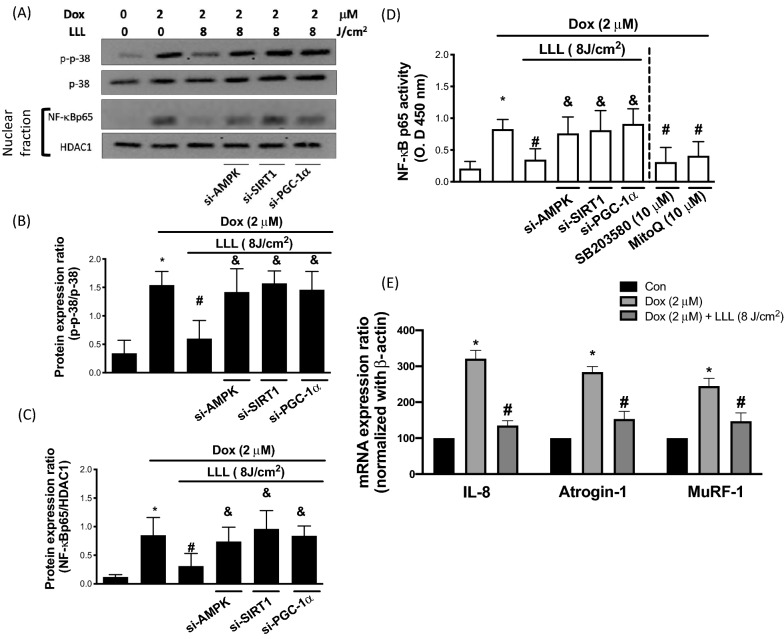
LLL exposure reduces Dox-caused proinflammatory responses by regulating AMPK/SIRT1/PGC-1a-mediated mitochondria function. C2C12 cells were treated with 2 µM doxorubicin (Dox) for a total of 24 h, in the LLL-treated group, cells were exposed to LLL 8 J/m^2^ before Dox treatment. In LLL plus AMPK, SIRT1, and PGC-1α silencing groups, cells were transfected with AMPK or SIRT1 or PGC-1α siRNA for 36 h before LLL exposure and Dox treatment. The expression level of p-p38, p38, nuclear NF-κBp65, and nuclear HADC1 were investigated using Western blot assay (**A**). Protein expression levels were quantified and presented by a bar chart (**B**, **C**). The activity of nuclear NF-κBp65 was investigated by a commercial kit (**D**). IL-8, Atrogin-1, and MuRF-1 mRNA expression levels were investigated by the real-time PCR. The data were presented as the mean ± SD of three biological replicates at three separate times. (* indicating p < 0.05 compared with the control group; # indicating p < 0.05 compared to Dox group; & indicating p < 0.05 compared to only LLL exposure group)

ROS have the capacity to evoke oxidative stress through the MAPK signaling pathway to activate NF-κB, which typically stays in the cytoplasm of nonstimulated cells. Upon stimulation, activation of NF-κB and nuclear translocation occur followed by inflammatory and apoptotic gene transcription. The involvement of P38 MAPK and NF-κB in Dox-evoked oxidative stress was determined to identify the intracellular pathways involved in the protective effects of LLL irradiation. The activation of P38 MAPK was estimated by measuring its phosphorylation level, and the activation of NF-κB was detected by measuring its content in the nuclear fraction. C2C12 cells were receiving LLL irradiation at an intensity of 8 J/m^2^ for 1 h before to exposure to 2 μM Dox for another 24 h. While LLL irradiation effectively inhibited the phosphorylation of P38 MAPK in Dox-stimulated C2C12 cells, we found that Dox notably increased the phosphorylation level of P38 MAPK. However, the beneficial effects of LLL irradiation were abolished in cells transfected with siRNA targeting AMPK, SIRT1, and PGC-1α (Fig. 6A–C). Additionally, the inhibitor of P38 (SB203580) and the mitochondrial antioxidant Mito Q effectively inhibited NF-kB activation caused by Dox, suggesting that Dox evoked NF-kB activation via the mitochondrial ROS-dependent and P38 MAPK pathways (Fig. 6D). The ubiquitin–proteasome system is one of the major pathways that regulate muscle protein degradation, and this system plays a central role in controlling muscle size. Atrogin-1 and MuRF-1 are two E3 ubiquitin ligases that are important regulators of ubiquitin-mediated protein degradation in skeletal muscles [7]. Consistent with prior research showing that activation of P38 MAPK induces the expression of Atrogin-1 [30] and MuRF-1 [15], our data indicated that Dox-induced transcription levels of Atrogin-1 and MuRF-1 as well as an inflammatory cytokine, IL-8, were significantly reduced in cells that had previously received LLL irradiation (Fig. 6E).

### LLL reduces Dox-induced skeletal muscle apoptosis by regulating SIRT1, AMPK, and PGC-1α

**Fig. 7:**
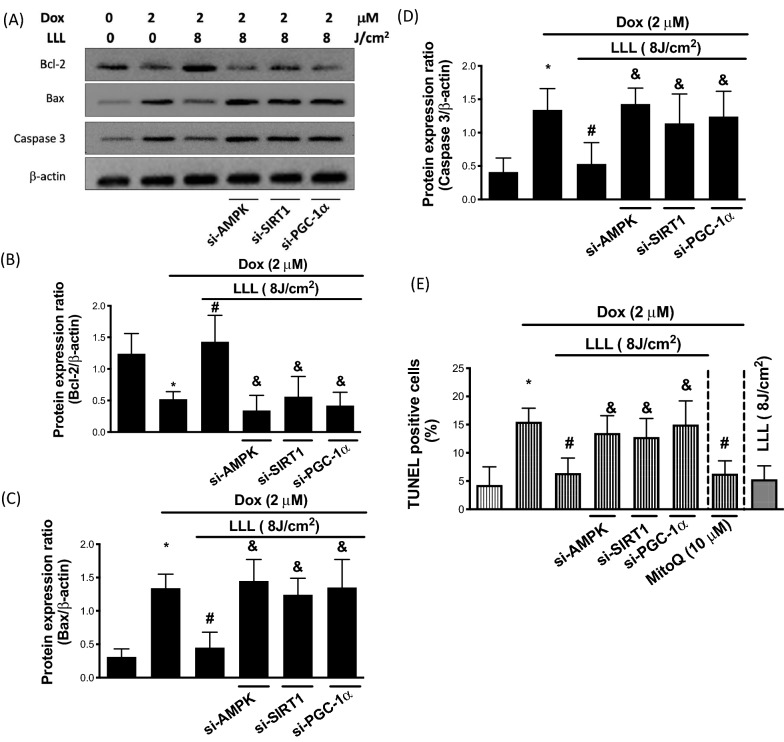
LLL exposure attenuates Dox-induced apoptosis. C2C12 cells were treated with 2 µM doxorubicin (Dox) for a total of 24 h, in the LLL-treated group, cells were exposed to LLL 8 J/m^2^ before Dox treatment. In LLL plus AMPK, SIRT1, and PGC-1α silencing groups, cells were transfected with AMPK or SIRT1 or PGC-1α siRNA for 36 h before LLL exposure and Dox treatment. The expression level of Bcl-2, Bax, caspase 3, and β-actin were investigated using Western blot assay (**A**). Protein expression levels were quantified and presented by a bar chart (**B**–**D**). The TUNEL assay was used for detecting apoptotic DNA fragmentation using flow cytometry. The data were presented as the mean ± SD of three biological replicates at three separate times. (* indicating p < 0.05 compared with the control group; # indicating p < 0.05 compared to Dox group; & indicating p < 0.05 compared to only LLL exposure group)

Dox has been previously demonstrated to be an inducer of apoptosis in C2C12 cell myotubes [31]. Mitochondrial dysfunction is a critical activator of proapoptotic events. Therefore, we examined skeletal muscle cell apoptosis to determine the beneficial effects of LLL irradiation in response to Dox. As such, the protein expression levels of Bax, Bcl-2, and caspase-3 were determined by Western blot assay. Our results showed that Dox increased the expression levels of Bax and caspase-3 but reduced the Bcl-2 levels, whereas LLL irradiation reversed these changes (Fig. 7A–D). The TUNEL assay was subsequently used to determine the number of apoptotic C2C12 cells by using flow cytometry. As shown in Fig. 7E, we found that LLL irradiation reduced the number of Dox-induced TUNEL-positive cells. In addition, skeletal muscle cells transfected with AMPK, SIRT1, and PGC-1α siRNA exhibited impaired protective effects of LLL on Dox-induced apoptosis, indicating that LLL downregulated Dox-induced skeletal muscle apoptosis via modulation of the AMPK/SIRT1/PGC-1α signaling pathway. Moreover, Dox-induced apoptosis was rescued in the presence of MitoQ, an antioxidant of mitochondria, which indicates the involvement of mitochondrial oxidative stress (Fig. 7E).

## Discussion

**Fig. 8:**
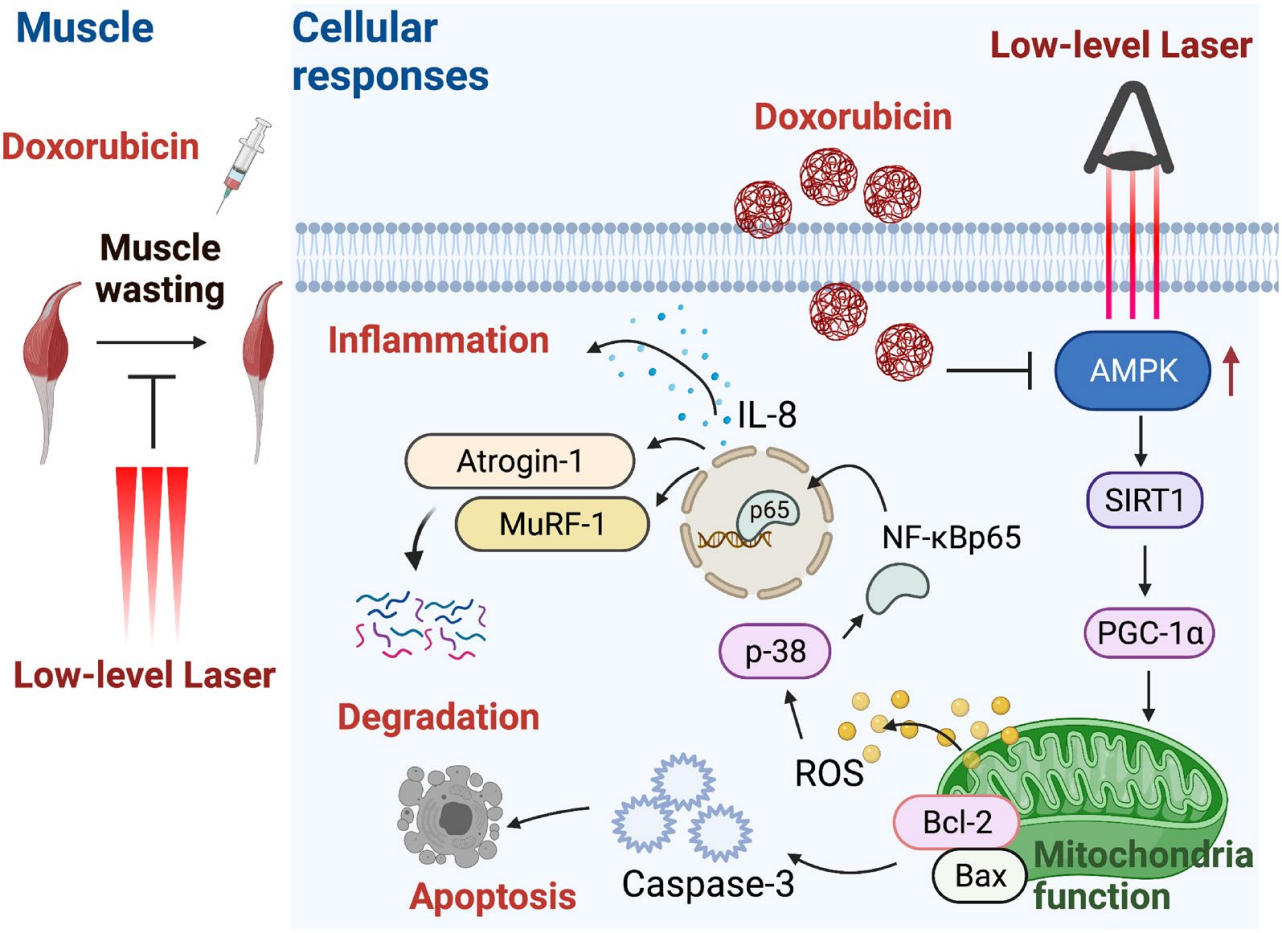
Schematic diagram summarizes the protective effects of LLL irradiation against Dox-induced skeletal muscle atrophy via the activation of AMPK/SIRT1/PGC-1α signaling. The in vivo and in vitro experiments show that LLL exposure promotes AMPK phosphorylation and upregulation of SIRT1 and PGC-1α expression thereby contributing to the preservation of mitochondrial homeostasis, and alleviation of oxidative stress and apoptosis in acute DOX treated skeletal muscles

Dox-induced mitochondrial dysfunction has become a pathological mechanism for the combined loss of muscle strength and constant discomfort of patients undergoing chemotherapy [6]. To our knowledge, this is the first study to elucidate the underlying cellular and molecular mechanisms by which LLL irradiation protects skeletal muscles against atrophy caused by Dox. Our main findings are as follows. (1) LLL irradiation suppresses Dox-induced muscle wasting in rats by diminishing ubiquitin-mediated protein degradation, (2) LLL irradiation restores Dox-reduced AMPK activation and downstream SIRT1 and PGC-1α, (3) LLL irradiation ameliorates Dox-impaired mitochondrial membrane potential and ROS generation, and (4) LLL diminishes ubiquitin-mediated protein degradation and apoptosis caused by Dox (Fig. 8).

Consistent with prior research that suggested that the activation of AMPK can enhance glucose uptake in isolated skeletal muscles [32] and that Dox-induced muscle atrophy can be attenuated by interventions that can activate the AMPK signaling pathway [33], the findings from the present study also suggested that cells exposed to LLL irradiation alone significantly increase the phosphorylation levels of AMPK and reverse Dox-impaired AMPK phosphorylation. SIRT1 is a key regulator of mitochondrial biogenesis and is known to activate PGC-1α by deacetylation in skeletal muscle cells [34]. The AMPK/SIRT1/PGC-1α signaling pathway acts as an energy-sensing network and plays a crucial regulatory role in mitochondrial biosynthesis, energy metabolism, and oxidative stress [11]. In the current study, our results indicated that LLL irradiation restores down-regulated SIRT1 and PGG-1α expression, suggesting that SIRT1/PGC-1α forms a regulatory axis to control mitochondrial function in Dox-induced myopathy. AMPK may also modulate downstream targets of SIRT1, including PGC-1α.

Mitochondria are the key source of intracellular ROS production and play a critical role in promoting skeletal muscle atrophy [35]. Targeting DNA is thought to be the principle cytotoxicity of Dox, and the covalently closed and circular characteristics of MtDNA are prone to intercalate with chemotherapies; thus, an increased possibility of transcriptional mistakes occurs, resulting in mitochondrial dysfunction [36]. In addition, prior research found mitochondria to be the major source of ROS formation in skeletal muscle in response to Dox [35]. Min et al. reported that a single injection of Dox alone at a dose of 20 mg/kg results in a decreased mitochondrial respiratory capacity and increased mitochondrial uncoupling and dysfunction in skeletal muscles [37]. Research has indicated that mitochondrial ROS stimulates protein degradation in muscles by activating proteolytic systems that include the ubiquitin–proteasome pathway and caspase cascades [16]. The results from the present study indicated that LLL irradiation preserved mitochondrial content, an effect accompanied by increased PCG-1α expression, which is a key regulator of energy metabolism upregulated in response to LLL irradiation. Accordingly, LLL may prevent Dox-induced atrophy through, at least in part, regulating mitochondrial dynamics to maintain mitochondrial content and function (Fig. 8).

Prior research also found that ROS-mediated P38 MAPK activation results in Dox-induced cardiomyopathy [38] and that oxidative stress-induced P38 MAPK activation is involved in cachectic muscle wasting through both ubiquitin–proteasome- and autophagy-lysosome-dependent proteolytic mechanisms [15]. Activation of P38 MAPK induces activation of agrogin-1 and MURF-1 expression in cachectic muscle wasting [15]. The results from the present study demonstrated that the beneficial effects of LLL irradiation protect skeletal muscle from Dox-induced atrophy via the P38 MAPK/NF-κB signaling pathway.

Additionally, LLL irradiation significantly ameliorated the apoptotic effect of doxorubicin by decreasing the Bax content and increasing the Bcl-2 content, which subsequently led to reduced caspase-3 activation in C2C12 cells treated with Dox. The strong anti-apoptotic effect of LLL irradiation is thought to be through, in part, the inhibition of P38 MAPK/NF-κB-mediated inflammation and apoptosis pathways, resulting in the prevention of all signals of cell death. This is similar to the results of a previous study reporting that LLL irradiation prevents the inflammatory cascade caused by TNF-α and thereby prevents the activation of proapoptotic protein-induced endothelial cell apoptosis [39].

## Conclusion

Collectively, our experiments provide in vivo and in vitro mechanistic evidence suggesting that LLL irradiation is a potent intervention against Dox-induced skeletal muscle atrophy via activation of AMPK/SIRT1/PGC-1α signaling. The results of this study may provide further insights into a possible molecular mechanism by which LLL irradiation protects against Dox-induced muscle atrophy, which may facilitate the development of adjuvant treatment.

## Supplementary Information

**Additional file 1. Table S1.** Primers in real time RT-PCR.

## Data Availability

The datasets generated and analyzed during the current study are available from the corresponding author on reasonable request.

## Abbreviations

Dox: Doxorubicin
LLLs: Low-level lasers
ATP: Adenosine triphosphate
AMPK: AMP-activated protein kinase
SIRT1: Sirtuin 1
PG-1α: Coactivator peroxisome proliferator-activated receptor coactivator-1α
FBS: Fetal bovine serum
JC-1: 5,58,6,68 Tetraethylbenzimidazolcarbocyanine iodide
