# Supplementary material for: Low-level laser prevents doxorubicin-induced skeletal muscle atrophy by modulating AMPK/SIRT1/PCG-1α-mediated mitochondrial function, apoptosis and up-regulation of pro-inflammatory responses
Source: Cell Biosci. 2021 Dec 7;11:200. doi: 10.1186/s13578-021-00719-w (PMC8650328; doi:10.1186/s13578-021-00719-w)
Supplement: Supplementary file 1 — Additional file 1. Table S1. Primers in real time RT-PCR. [file 13578_2021_719_MOESM1_ESM.docx]

**Additional file 1: Table S1.** Primers in real time RT-PCR

| Target gene | Forward | Reverse |
| --- | --- | --- |
| Atrogin-1 | 5’-CAGAGAGGCAGATTCGCAAG-3’ | 5’-GGTGACCCCATACTGCTCTC-3’ |
| MuRF1 | 5’- TGCCTACTTGCTCCTTGTGC-3’ | 5’- CACCAGCATGGAGATGCAGT-3’ |
| IL-8 | 5’- GTGCAGTTTTGC CAAGGAGT-3’ | 5’- TTATGAATTCTCAGCCCT CTTCAAAAACTTCTC-3’ |
| β-actin | 5’- CATTGCTGACAGGATGCAGAAGG-3’ | 5’- TGCTGGAAGGTGGACAGTGAGG-3’ |
